# Spinal Cord Infarction as a Cause of Acute Myelopathy

**DOI:** 10.7759/cureus.50379

**Published:** 2023-12-12

**Authors:** Ahmed Harazeen, Anand Patel, Chilvana Patel

**Affiliations:** 1 Neurology, Emory University School of Medicine, Atlanta, USA; 2 Neurology, Baylor College of Medicine, Houston, USA; 3 Neurology, University of Texas Medical Branch, Galveston, USA

**Keywords:** acute bilateral weakness, spinal cord ischemia, myelopathy, acute stroke, spinal cord infarct

## Abstract

Spinal cord infarction is an uncommon and often perplexing condition for emergency doctors to diagnose. Its initial symptoms are general and non-distinct, leading to frequent misdiagnosis. This case report is about a 56-year-old woman who presented to the hospital with substernal tightening chest pain and rapidly progressing bilateral lower-extremity weakness. Initially, she was diagnosed with spinal cord infarction based on magnetic resonance imaging (MRI) and cerebrospinal fluid (CSF) studies, with all other differential diagnoses ruled out. This article explores the utility of advanced MRI techniques, particularly diffusion-weighted imaging (DWI) sequence, in diagnosing spinal cord infarction. This is especially pertinent in patients who present with atypical symptoms and do not have conventional risk factors for spinal cord ischemia.

## Introduction

Acute spinal cord stroke (ASCS) is a rare, yet serious condition that occurs when the blood supply to the spinal cord is suddenly interrupted [1]. The spinal cord plays a crucial role in the central nervous system, transmitting sensory and motor signals between the brain and the peripheral body parts as well as facilitating complex reflex actions that occur without direct brain involvement. ASCS can result in various neurological deficits, including sensory and motor impairment, and bowel or bladder dysfunction. SCS arises from ischemia, which occurs due to blockages or embolisms in the spinal arteries. Dissection, chronic inflammatory conditions, or compression of the spinal cord can also cause ASCS. The clinical presentation of ASCS varies depending on the location and extent of the injury [2].

## Case presentation

A 56-year-old female with no past medical history presented to the hospital complaining of a one-day history of substernal chest pain, abdominal cramps, bilateral lower-extremity weakness, and urinary incontinence. Initial examination revealed paraparesis with at least 3/5 strength in her bilateral lower extremities and intact reflexes, sensation, coordination, and cranial nerves exam. However, symptoms rapidly progressed early in the hospitalization course. On day 3, the exam showed decreased strength 0/5 proximally and 3/5 distally in both legs, T5 sensory level, absent reflexes at the knees and ankles, constipation, and urinary retention. Otherwise, mental status and cranial nerves remained intact. The initial laboratory exam was unremarkable for any cardiac etiology with negative troponin and EKG as workup for chest pain.

**Figure 1:**
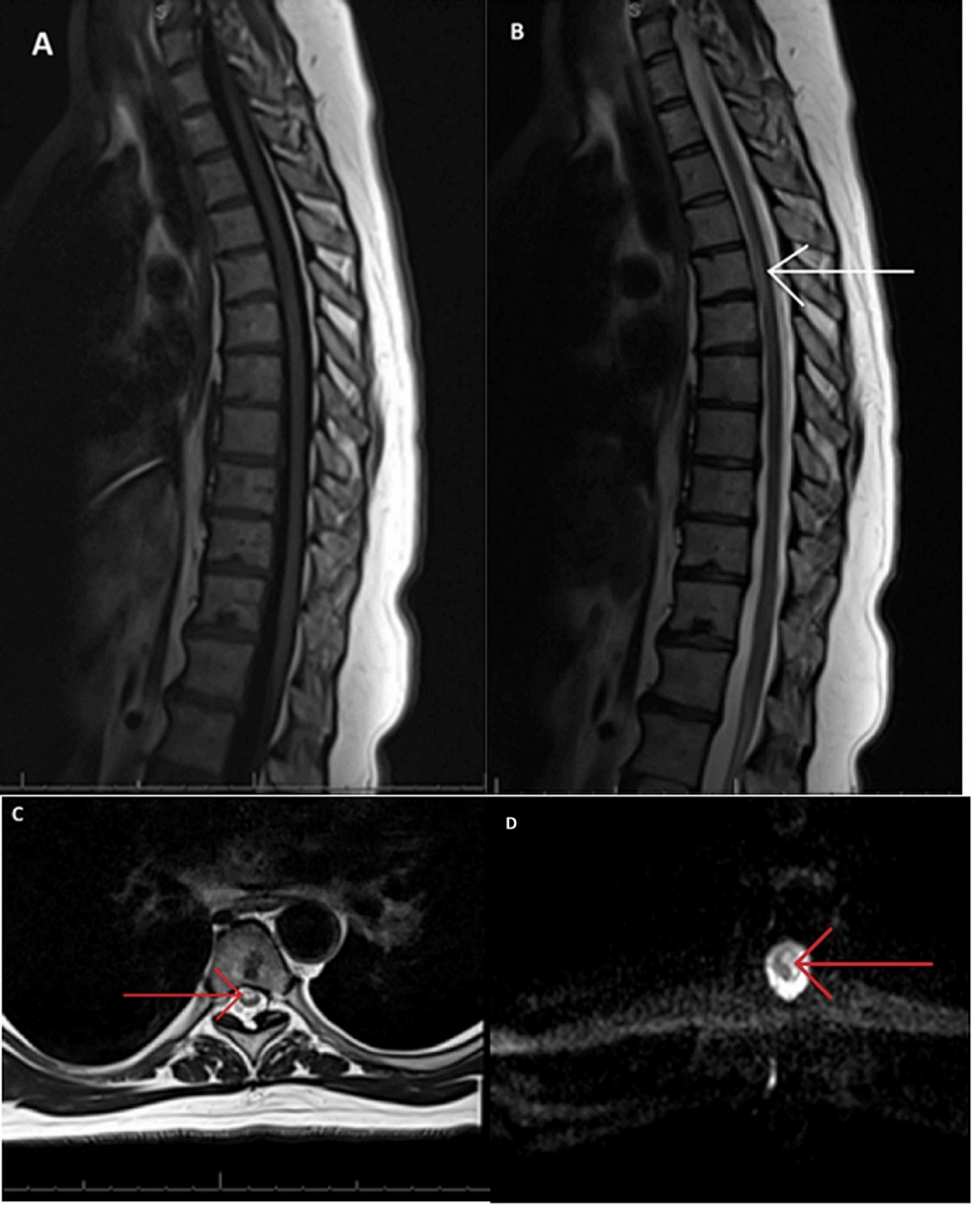
Magnetic resonance imaging of the thoracic spine. A) Sagittal T1 sequence was unremarkable. B) T2 sequence showing non-enhancing, linear hyperintense signal in the anterior bilateral spinal cord from T3-T5 without appreciable cord expansion or atrophy. C) Axial T2 sequence at T4 showing hyperintense signal in the anterior bilateral spinal cord. D) Axial diffusion-weighted imaging sequence at T4 showing diffusion restriction in the spine.

MRI of her spine revealed a non-enhancing, linear T2-hyperintensity signal in the anterior spinal cord bilaterally extending from T3-T5 levels without cord expansion or atrophic changes and diffusion-weighted imaging (DWI) sequence at T4 showing diffusion restriction (Figure 1). Cerebrospinal fluid (CSF) studies were significant for elevated protein at 120 mg/dL (normal values from 15 to 60 mg/dL) in the presence of clear fluid and normal glucose and white blood cells. The serologic study was negative for erythrocyte sedimentation rate, C-reactive protein, cryoglobulins, human immunodeficiency virus, hepatitis C virus, syphilis, antinuclear antibodies, Sjogren's anti-SS-A, SS-B, anti-double-stranded deoxyribonucleic acid antibodies, antineutrophilic cytoplasmic antibody, anti-aquaporin-4 IgG, factor V Leiden, and angiotensin-converting enzyme. The nutritional evaluation was unremarkable without evidence of vitamin deficiency such as vitamin B1, B6, B12, or D. Hypercoagulability studies including anticardiolipin antibodies were unremarkable.

The initial presentation and subsequent imaging were concerning for spinal cord infarction (SCI) along the course of the anterior spinal artery in the absence of precipitating factors. CT angiography revealed a normal-caliber thoracoabdominal aorta without evidence of compression, dissection, atherosclerosis, or plaques, an incidental acute pulmonary embolism, and incidental multiple thyroid nodules. A transthoracic echocardiogram showed normal systolic and diastolic function with an incidental large interatrial shunt. No evidence of deep or superficial venous thrombosis on the lower-extremity duplex. Her hospitalization was complicated by a urinary infection with extended-spectrum beta-lactamase requiring multiple antibiotic treatments. The patient was discharged on apixaban for six months to an inpatient rehabilitation facility during which her strength continued to improve with unchanged sensory or bladder symptoms.

The patient was seen after three months in the outpatient and she was complaining of severe neuropathic pain, muscle spasms, multiple skin ulcers, and urinary incontinence. 

## Discussion

In this case report, we have explored a rare instance of spontaneous SCI in a 56-year-old female, presenting an opportunity to discuss the broader context of spinal cord syndromes and their diagnostic challenges. Spinal cord syndromes, which include anterior cord, posterior cord, and Brown-Sequard syndrome, are characterized by distinct clinical and anatomical features. Notably, anterior cord syndrome manifests with symptoms such as loss of pain and temperature sensation, flaccid paralysis, and areflexia below the level of injury, while preserving touch and proprioception [1].

The vascular anatomy of the spinal cord is facilitated by three major arteries. The anterior spinal artery, originating from the vertebral arteries, supplies the anterior two-thirds of the spinal cord. This artery gives rise to the sulcal artery, responsible for nourishing the gray matter [2]. The posterior section of the spinal cord receives blood from two posterior spinal arteries, which predominantly stem from the posterior inferior cerebellar artery. Additionally, the arterial vasocorona, connecting the anterior and posterior spinal arteries, ensures the lateral part of the spinal cord is well supplied. Segmental medullary arteries, branching from various sources depending on their spinal location, play a crucial role in providing blood to the spinal cord and vertebral bone. Notably, the artery of Adamkiewicz, predominantly found on the left side, emerges as the most significant medullary artery, originating between T8 and T12 levels [3]. 

The etiology of spontaneous SCI varies, ranging from embolic events and aortic atherosclerosis to rarer conditions like surfer's myelopathy and fibrocartilaginous emboli. In our patient, the absence of common precipitating factors such as aortic dissection, which typically requires urgent intervention, made the diagnosis particularly challenging [4]. This is reflective of the broader clinical challenge in SCI, where 20-40% of cases do not have an identifiable cause [4-7].

In this context, the role of advanced imaging techniques becomes paramount. DWI in MRI played a crucial role in our diagnosis, given its sensitivity to changes in water diffusion in spinal cord tissue. DWI sequence measures the random movement of water molecules in tissue by applying gradients to the magnetic field, which causes the molecules to move in a particular direction. DWI can detect acute pathological changes such as edema, necrosis, and inflammation, which might not be visible on other imaging sequences [4,5]. Our patient's MRI findings, particularly on DWI, were instrumental in excluding compressive myelopathy and confirming SCI, thereby exemplifying the critical role of advanced imaging in the diagnosis of atypical SCI presentations.

Conclusively, the diagnosis of idiopathic spontaneous SCI, as illustrated in our case, requires a meticulous, exclusionary approach. This involves a comprehensive evaluation, detailed history-taking, comprehensive physical examination, and extensive investigations to rule out infectious, inflammatory, and vascular causes. Although SCI is largely a diagnosis of exclusion, the use of advanced imaging modalities, especially MRI with DWI, has emerged as a crucial tool in diagnosing and understanding this complex condition [8].

## Conclusions

This case report elucidates a rare instance of SCI in a 56-year-old female, underscoring the diagnostic challenges and the necessity for a high index of suspicion in cases of acute myelopathy with atypical presentations. The rapid progression of symptoms from chest pain to severe paraparesis and autonomic dysfunction, coupled with diagnostic findings from MRI and DWI, was pivotal in identifying SCI in the absence of common risk factors or identifiable causes. This highlights the importance of considering SCI in differential diagnoses, especially when patients present with non-specific or rapidly progressing neurological deficits.

The patient's subsequent complications, including neuropathic pain, muscle spasms, and urinary incontinence, further emphasize the long-term impact of SCI and the need for comprehensive, multidisciplinary management. This case contributes to the limited literature on spontaneous SCI, providing insights into its potential presentation, the critical role of advanced imaging in the diagnosis, and the importance of considering it as a differential in cases of unexplained acute myelopathy. It reinforces the need for continued research and awareness to improve diagnostic accuracy and patient outcomes in this challenging and often debilitating condition.
